# New Insights Into the Persistent Effects of Acute Exposure to AFB_1_ on Rat Liver

**DOI:** 10.3389/fmicb.2022.911757

**Published:** 2022-06-16

**Authors:** Jiahui Yan, Lin Chen, Li Zhang, Zhaohuan Zhang, Yong Zhao, Yuan Wang, Jie Ou

**Affiliations:** ^1^College of Food Sciences and Technology, Shanghai Ocean University, Shanghai, China; ^2^Engineering Research Center of Modern Preparation Technology of TCM, Shanghai University of Traditional Chinese Medicine, Shanghai, China; ^3^Shanghai Engineering Research Center of Aquatic Product Processing & Preservation, Shanghai, China; ^4^Laboratory of Quality and Safety Risk Assessment for Aquatic Product on Storage and Preservation, Ministry of Agriculture and Rural Affairs, Shanghai, China

**Keywords:** aflatoxin B_1_, RNA-seq, hepatotoxicity, target gene, oxidative stress

## Abstract

Aflatoxin B_1_ (AFB_1_) has mutagenesis, carcinogenesis and teratogenesis effects and mainly found in food crops and their processed foods. AFB_1_ exposure can cause acute or chronic liver poisoning, but there were few studies on the persistent effects of acute AFB_1_ exposure on the liver. In this study, rat liver injury models were established 2 and 7 days after single exposure to high and low doses of AFB_1_. The persistent effects of AFB_1_ single acute exposure (ASAE) on rat liver were analyzed from the phenotypic and genetic levels. The results showed that compared with the control group, liver function indexes, MDA content in liver and the number of apoptotic hepatocytes in model groups increased to the highest on the 2nd day after ASAE (*p* < 0.001). However, the changes of liver coefficient were most significant on the 7th day after ASAE (*p* < 0.01). The results of liver pathology showed that the liver injury was not alleviated and the activities of antioxidant enzymes GSH-Px and SOD were the lowest on the 7th day (*p* < 0.001). RNA-Seq results indicated that there were 236, 33, 679, and 78 significantly differentially expressed genes (DEGs) in the model groups (LA-2d, LA-7d, HA-2d, HA-7d) compared with the control group. Among them, the *Gtse1* gene related to the proliferation, differentiation and metastasis of liver cancer cells, the *Lama5* and *Fabp4* gene related to the inflammatory response were significantly DEGs in the four model groups, and the differential expression of the immune system-related Bcl6 gene increased with the prolonged observation time after ASAE. In conclusion, ASAE can cause persistent liver damage in rats. The persistently affected genes *Lama5*, *Gtse1*, *Fabp4*, and *Bcl*6 possess the potential to be therapeutic targets for liver disease induced by AFB_1_.

## Introduction

Aflatoxins (AFs) is a general term for a class of highly toxic secondary metabolites produced by *Aspergillus parasiticus* and *Aspergillus flavus*. Crops such as grain and oilseed are easily contaminated by AFs during planting, harvesting, storage and processing. In addition, its physical and chemical properties are stable. Therefore, there is a great risk of harming animal and human health through the food chain (Rushing and Selim, 2019). At present, more than 20 kinds of AF have been found, among which AFB_1_ is the most toxic and most common, and the target organ of its toxic effect is mainly the liver (Hussein and Brasel, 2001). A warm and humid environment is easy to cause a large amount of AFB_1_ pollution (Abrar et al., 2013), so acute or chronic poisoning is often caused by dietary exposure to AFB_1_. Table 1 lists the effects of different doses of AFB_1_ and modeling time on the liver in some studies. It can be seen from the table that AFB_1_ can cause acute or chronic poisoning in the liver. But viewed from the model, there are few studies on the lasting effects of AFB_1_ exposure on the liver.

**TABLE 1:** Different animal models were established to explore the effects of AFB_1_ on liver.

Species	Dose	Modeling time (d)	Breeding time after modeling (h)	Effect (liver injury)	References
Rat	2 mg/kg	1	48	Acute	Deng et al., 2020
Rat	1 mg/kg	1	96	Acute	Monmeesil et al., 2019
Mice	3 mg/kg	2	12	Acute	Ishida et al., 2020
Mice	100 μg/day	14	12	Chronic	Long et al., 2016
Mice	0.75 mg/kg	30	24	Chronic	Xu et al., 2019
Rat	20 μg/day	42	24	Chronic	Nayak and Sashidhar, 2010
Rat	25 μg/day	90	24	Chronic	El-Agamy, 2010

AFB_1_ is metabolized by the P450 enzyme system in the liver into the ultimate carcinogen aflatoxin B_1_-8, 9-epoxide (AFBO), which has strong oxidation ability and can induce the body to produce a large amount of reactive oxygen species (ROS) (Guengerich et al., 1998). AFBO can covalently bind to DNA guanine N7 to form adduct AFB_1_-N7-guanine (Lin et al., 2014), and oxidize guanine to produce DNA damage marker 8-OH-deoxyguanine (Bailey et al., 1996; Wang and Groopman, 1999). In addition, excessive ROS will break the dynamic balance of the redox system and cause oxidative stress reaction. It also attacks cells, causing cells damage and inducing apoptosis. Therefore, this study explored the liver injury in model rats by analyzing the changes of phenotypic indicators related to oxidative stress response.

RNA-seq is a widely used parallel sequencing method. Through this technology, the complete transcript of the test sample and its expression level can be found (Wang et al., 2009). From the obtained data, we can not only know individual genes but also understand the functional pathways of gene enrichment through the analysis of functional database. RNA-seq data has been used to analyze the underlying mechanism of carotid atherosclerosis (CAS). Differential and functional enrichment analysis of sequencing data from CAS patients and control group showed that inflammation and immune response were the potential pathogenesis of CAS, and significant DEGs *CCR5*, *NPY*, and *NPY5R* were potential therapeutic targets of CAS (Li et al., 2021). The method is also often used to study cancer therapeutic targets. Bioinformatics analysis of DEGs in ovarian cancer and normal tissue showed that they were mainly concentrated in metabolic, cell cycle regulation and antibiotic biosynthesis pathways. Meanwhile, 10 central genes including *CCNB2*, *TYMS* and *KIF11* were analyzed (Yang et al., 2020). Sorafenib is the only FDA approved oral multi-target tyrosine kinase inhibitor that promotes apoptosis, reduces angiogenesis and inhibits tumor cell proliferation in the treatment of liver cancer. However, drug resistance and side effects of varying degrees were found in the process of drug use (Trojan and Zeuzem, 2013; Rota Caremoli and Labianca, 2014). Therefore, more potential genes for targeted treatment of liver diseases can be searched through gene sequencing technology.

This paper explores the sustained effects of AFB_1_ on the rat liver by establishing a model of ASAE. Based on this model, the RNA-Seq data were analyzed to find potential target genes for the treatment of liver diseases caused by AFB_1_, providing a new research basis for the development of targeted therapy for liver diseases.

## Materials and Methods

### Chemicals

Aflatoxin B_1_ (≥ 99%) and Dimethyl sulfoxide (DMSO) were purchased from Shanghai Acmec Biochemical Co., Ltd., and Sangon Biotech Co., Ltd. (Shanghai, China), respectively. Assay kits for the measurements of malondialdehyde (MDA), superoxide dismutase (SOD) Glutathione peroxidase (GSH-Px) and total protein (TP) were purchased from Nanjing Jiancheng Bioengineering Institute (Nanjing, China). Assay kits for the total RNA extraction, PowerUp SYBR Green Master Mix (Thermo Fisher Scientific, America) and HiScript III-RT SuperMix for qPCR (Vazyme, China).

### Animals

A total of 36 male Wistar rats were purchased from Shanghai SLAC Laboratory Animal Co., Ltd. Body weight (BW): 180-200 g. All rats were housed in an environmentally controlled room at 22-24°C with a relative humidity of 50-55% under a cycle of 12 h each light/dark. Food and water were available *ad libitum*. All animal procedures were performed in accordance with the Guidelines for Care and Use of Laboratory Animals of Shanghai University of Traditional Chinese Medicine and approved by the Animal Ethics Committee of Animal Center of China and Shanghai University of Traditional Chinese Medicine (Ethics number: PZSHUTCM210115014).

### Experimental Design

After one week of adaptive feeding, 36 rats were randomly divided into 3 groups (12 in each group): blank group (CK): normal breeding; AFB_1_ high-dose (HA) group: AFB_1_ was intraperitoneal injection with a dose of 2 mg/kg BW, only once; AFB_1_ low-dose (LA) group: AFB_1_ was intraperitoneal injection at a dose of 1 mg/kg BW, only once (AFB_1_ soluble in 4% DMSO). The experimental doses of AFB_1_ were determined according to references and results of pre-experiment (Monmeesil et al., 2019; Deng et al., 2020).

The above dose groups were determined based on references and preliminary experiments. The concentration of DMSO did not affect the normal growth of rats (Chen et al., 2020). Blood samples were collected from ocular venous plexus at the 1st, 4th and 7th day after ASAE and serum were prepared. On the 2nd day after ASAE, 6 rats in each group were randomly selected to weigh their final body weight. The rats were anesthetized with 10% chloral hydrate (350 mg/kg). After anesthesia, the rats were dissected, the blood of abdominal aorta was taken, the liver was separated, washed with normal saline, sucked dry and weighed. Liver tissues of an appropriate size were taken from the same part of the liver of each rat and soaked in 4% paraformaldehyde solution, which was fully fixed for histopathological study, and the rest tissues were frozen in liquid nitrogen and stored in a cryogenic refrigerator at −80°C for use. The remaining 6 rats in each group were treated with the same method on the 7th day after ASAE.

### Hepatic Pathological Examination

After being fixed in 4% paraformaldehyde for 24 h, the liver was removed and dehydrated in graded alcohol series, transparent in xylene. Tissues were embedded in paraffin, and paraffin blocks were then cut to 4 μm thickness using a microtome (RM2016, Shanghai Leica Instrument Co., Ltd., Germany). These sections were deparaffinized, rehydrated and stained using hematoxylin-eosin (HE) and then analyzed by optical microscopy (Nikon Eclipse E100, Japan) to evaluate histopathological changes. Observe the morphologies of the liver and spleen tissues were observed at 100 ×.

### Determination of Biochemical Indices in Serum and Liver

The serum was separated and measured the levels of alanine aminotransferase (ALT), alkaline phosphatase (ALP), aspartate aminotransferase (AST) and total bilirubin (TBIL) by automated chemistry analyzer (ADVIA 2120i, Hitachi, Ltd., Japan). The activities of SOD and GSH-Px, as well as the level of MDA were examined using commercial assay kits.

### Determination of Hepatocyte Apoptosis Rate

The apoptosis rate of rat hepatocytes in the model groups were detected by TUNEL method. Paraffin sections were taken in sequence for deparaffinize and rehydrate, antigen retrieval, permeabilization, equilibration at room temperature, TUNEL reaction, BSA blocking, addition of double antibodies, DAPI counterstain in nucleus and then sealed for microscopic examination. Stained nuclei were blue under UV excitation and positive apoptotic cells were green. The number of apoptotic hepatocytes in the total field of view was counted as well as the total number of hepatocytes. Calculation formula: Hepatocyte apoptosis rate = (Number of apoptotic hepatocytes/Total number of hepatocytes) × 100%.

### Illumina RNA Sequencing

Total RNA in the rats’ liver was extracted using the MagMAX™ mir Vana™ Total RNA Isolation Kit following the specification (Thermo Scientific™ KingFisher™ Flex™, Finland). Total RNA of each sample was quantified and qualified by Agilent 2100/2200 Bioanalyzer (Agilent Technologies, Palo Alto, CA, United States), NanoDrop (Thermo Fisher Scientific Inc.). 1 μg total RNA was used for following library preparation. Illumina RNA sequencing experiments used three parallel samples. The sequencing library construction and Illumina sequencing were conducted at GENEWIZ.

### Real-Time Quantitative PCR Analysis

To verify the reliability of the expression profiles observed in the RNA-Seq data, four genes were selected for real-time quantitative PCR (Q-PCR) analysis with the same experimental samples as in the RNA-Seq experiments. Total RNA was extracted using a King Fisher Flex automated nucleic acid extractor and accompanying kit, and reverse transcribed after RNA electrophoresis to determine RNA quality. β*-Actin* was used as an internal reference and primers designed by Primer 5 are shown in Table 2, and relative expression was calculated according to the 2^–ΔΔct^ relative quantification formula.

**TABLE 2:** Primer information.

Gene	Primer sequence (5′to′)	Number of bases
β*-Actin*	Forward: AGCGTGGCTACAGCTTCACC	20
	Reverse: AAGTCTAGGGCAACATAGCACAGC	24
*Lama5*	Forward: TTCACAGGAGAGCGGTGTTC	20
	Reverse: CGTTGCAGTCACAGTTCACG	20
*Ccnd1*	Forward: TCAAGTGTGACCCGGACTG	19
	Reverse: GACCAGCTTCTTCCTCCACTT	21
*Cdkn1a*	Forward: TGTGATATGTACCAGCCACAGG	22
	Reverse: GCGAAGTCAAAGTTCCACCG	20
*Gpx2*	Forward: ATCAGTTCGGACATCAGGAGA	21
	Reverse: TCACCATTCACCTCGCACTT	20

### Statistical Analysis

Results were analyzed using GraphPad Prism 8.0 (GraphPad Software, La Jolla, California), SPSS 25 (IBM, NY, United States) and Origin 8.0 software (Origin Inc., Northampton, MA, United States). Analysis of variance (ANOVA) was performed followed by Tukey’s test with a confidence interval of 95% (*p* ≤ 0.05).

## Results

### Body Weight and Liver Index Changes in Rats

**FIGURE 1:**
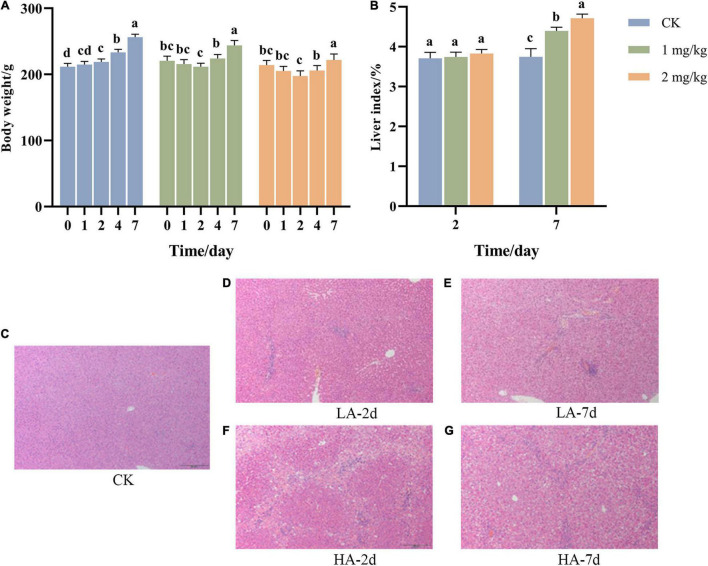
Changes of **(A)** body weight, **(B)** liver index, and **(C–G)** liver pathological morphology with time after ASAE in rats (HE, × 100). 0: weight before ASAE; 1 and 4: weight on 1st and 4thr days after ASAE; 2 and 7: weight and index on 2nd and 7th days after ASAE. LA-2d and HA-2d: rats were sacrificed on the 2nd day after ASAE. LA-7d and HA-7d: rats were sacrificed on the 7th day after ASAE. The data are expressed as mean ± standard deviation. Different letters representing significant difference (*p* < 0.05) (the same below).

During the experiment, the BW of the rats was weighed before ASAE and on the 1st, 2nd, 4th and 7th after ASAE (Figure 1A). The BW of rats in the CK group showed an upward trend in the whole experimental cycle, while that in the model groups decreased and then increased.

The organ index is one of the main indicators of the biological properties of animals (Zou et al., 2010). Figure 1B shows that there was no significant difference in liver index between rats treated on the 2nd day after ASAE and the CK group. Compared on the 7th day after ASAE with the CK group, the liver coefficient of the model groups was significantly higher (*p* < 0.05), and the HA group was significantly different from the CK group (*p* < 0.001).

### Histopathological Changes of Liver Tissue in Rats

Representative hepatic histopathological examination results of each group were shown in Figures 1C–G. The rats in the CK group had a normal liver lobular architecture and cell structure. The nucleolus was clear, homogenous cytoplasm (Figure 1C). Livers illustrated different degrees of pathological changes in the model groups. In LA-2d group, the liver exhibited moderate spotty necrosis and degeneration (Figure 1D). In LA-7d group, moderate spotty necrosis, vacuolar degeneration, and mild inflammatory cell infiltration were observed in the portal area (Figure 1E). Hepatocyte lytic necrosis, called hepatocyte bridging necrosis, was widely found in the liver pathological sections of rats in HA-2d group. This phenomenon was common in liver poisoning or severe chronic hepatitis (Figure 1F). In HA-7d group, in addition to severe bridging necrosis of hepatocytes, there were also vacuolar degeneration of hepatocytes and fibrous tissue hyperplasia (Figure 1G).

### Effect of AFB_1_ on Liver Function

**FIGURE 2:**
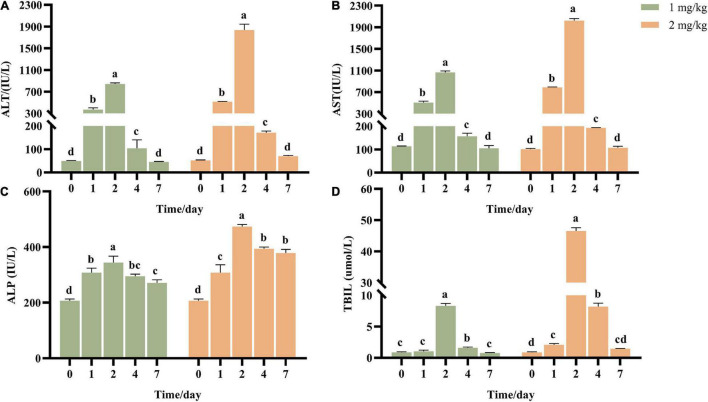
Effect of AFB_1_ on serum liver function indicators in rats. **(A)** Serum alanine aminotransferase (ALT), **(B)** aspartate aminotransferase (AST), **(C)** alkaline phosphatase (ALP) activities, and **(D)** total bilirubin (TBIL) were measured. 0: serum samples before ASAE; 1, 2, 4 and 7: serum samples on 1st, 2nd, 4th and 7th days after ASAE.

Transaminase ALT and AST are common indicators to determine whether the liver is damaged. ALT mainly exists in the plasma of hepatocytes, and the elevation of ALT in serum reflects the injury of hepatocytes. AST mainly exists in mitochondria of hepatocytes, and elevated AST indicates more serious liver injury. ALP and TBIL are the detection indexes reflecting hepatic bile metabolism. After ASAE, ALT, AST, ALP and TBIL in the model groups increased first and then decreased (Figure 2). It reached the peak on the 2nd day after ASAE, which was significantly different from that in the CK group (*p* < 0.001). Except ALP, other indexes returned to normal level on the 7th day after ASAE.

### Analysis of Apoptosis in Rat Hepatocytes

**FIGURE 3:**
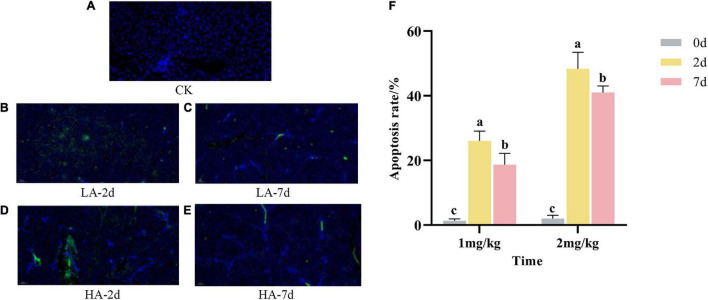
Effect of AFB_1_ on apoptosis in rat hepatocytes. **(A–E)**: representative images of TUNEL staining (× 200), **(F)**: TUNEL assay for percentage of hepatocyte apoptosis.

Apoptosis refers to the process by which cells are affected by abnormal factors and controlled by genes to end their life autonomously. As shown in Figures 3A-E, the nuclear membranes of normal hepatocytes were intact and stained blue; the nuclear membranes of apoptotic hepatocytes were clustered or fragmented and stained green. As can be seen from Figure 3F, compared with the CK group, the apoptosis rate in the model groups was significantly increased in a dose-dependent manner. However, the apoptosis rate of hepatocytes on the 7th after ASAE was slightly lower than that on the 2nd after ASAE, and there was still significant difference (*p* < 0.05).

### Hepatic Antioxidative Status and Lipid Peroxidation

**FIGURE 4:**
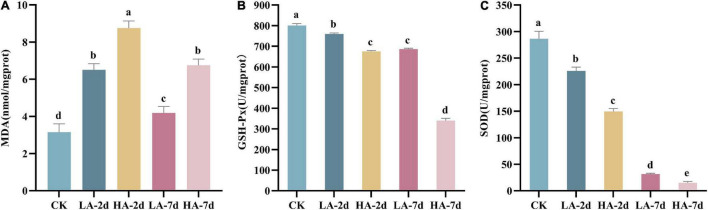
Effect of AFB_1_ on hepatic tissue lipid peroxidation level and antioxidant enzyme activities in rats. **(A)** Hepatic malondialdehyde (MDA) content, **(B)** hepatic glutathione peroxidase (GSH-Px) and **(C)** hepatic superoxide dismutase (SOD) activity.

The content of MDA can reflect the degree of lipid peroxidation and indirectly reflect the degree of cell damage. In this study, the MDA content in model groups were significantly higher than that in CK group (*p* < 0.05) (Figure 4A), indicating that AFB_1_ caused hepatocyte injury. It was also found that MDA content in the liver of the rats after ASAE not only showed a dose-dependent manner, but also first increasing and then decreasing trend with prolonged observation. However, it was still significantly higher than the CK group and did not return to the normal levels on the 7th day (*p* < 0.05).

Studies have shown that AFB_1_ can lead to the imbalance of the body’s oxidative system homeostasis and cause oxidative stress response. The antioxidant enzymes SOD and GSH-Px are the body’s first line of defense against free radicals. In this study, compared with the CK group, the activities of SOD and GSH-Px in the model groups decreased significantly (*p* < 0.05) (Figures 4B,C). And with the prolonged observation time after ASAE, the activities of SOD and GSH-Px became lower and lower (*p* < 0.001).

### Differential Expression Genes and Functional Analysis

**FIGURE 5:**
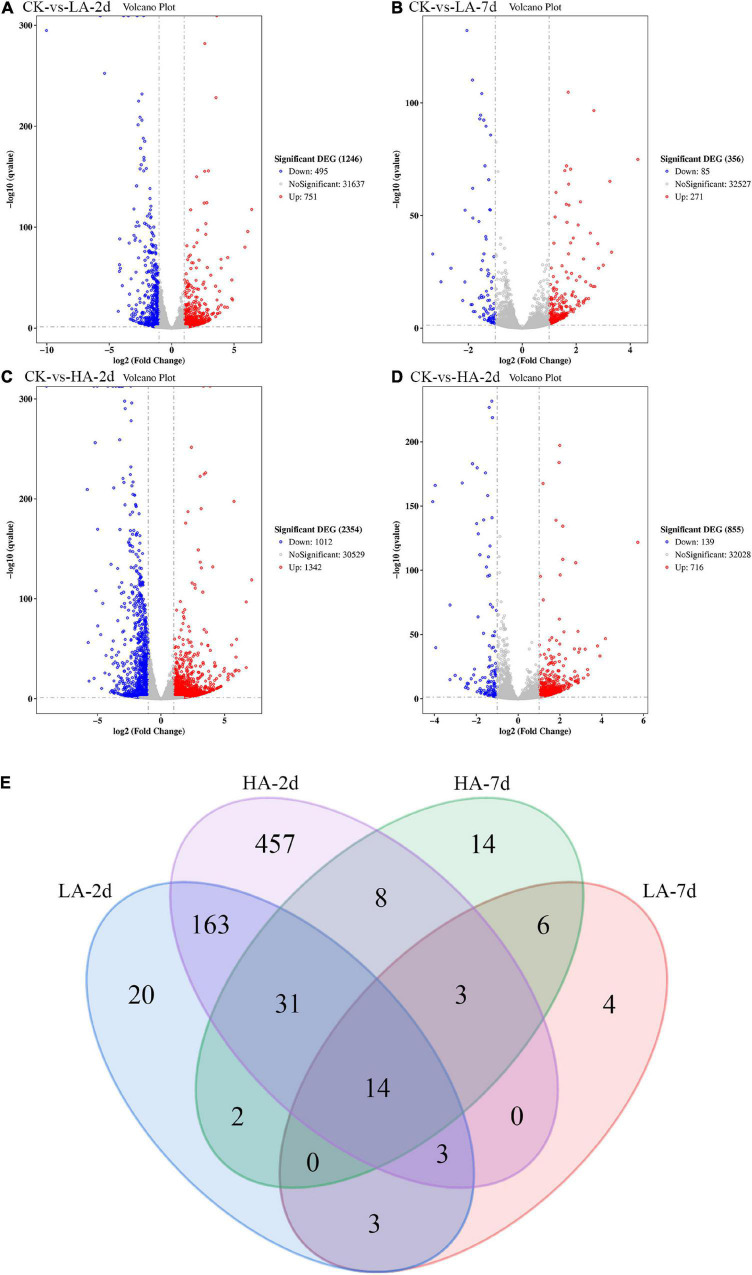
The Volcano plot and Venn diagram of DGEs. **(A–D)** Red spots indicate significantly up-regulated genes; blue spots indicate significantly down-regulated genes; gray spots are genes of no differential expression. The horizontal line at false discovery rate (FDR) = 0.05; vertical line at | log2FC| = 1. **(E)** There were special DEGs in the different analyses of each group (Venn diagram, FDR ≤ 0.01 and | log2FC| ≥ 2).

In this study, the DEGs between the model groups and the CK group were obtained by RNA-Seq technology, and a volcano map was drawn (Figures 5A–D). The volcano plot showed that LA-2d, LA-7d, HA-2d and HA-7d groups had 1246, 356, 2354, and 855 DEGs, respectively (FDR ≤ 0.05 and | log_2_FC| ≥ 1). From this result, it can be found that the number of DEGs was related to the dose of ASAE and the observation time after ASAE: the higher the dose, the more DEGs; the number of DEGs decreased with the prolonged time of continuous impact. Table 3 shows the classification and number of entries of KEGG pathways enriched in each group of DEGs. Looking at the total number of KEGG pathways in each model group, there were fewer on the 7th day after ASAE than on the 2nd day. The number of entries for each classification in each group shows that the main difference was in the metabolic category. This is related to the fact that the liver is the largest metabolic organ of the body as well as being extremely self-repairing.

**TABLE 3:** Number of pathways for each KEGG classification in the model group.

Level	LA-2d	LA-7d	HA-2d	HA-7d
Cellular Processes	22	20	22	21
Environmental Information Processing	33	32	34	33
Genetic Information Processing	13	13	16	14
Human Diseases	84	75	84	82
Metabolism	83	48	91	67
Organismal Systems	84	74	85	84
**Total**	320	262	333	301

In order to explore the genes that were continuously affected and their functions, the screening criteria were strengthened, and the Venn diagram as shown was drawn (FDR ≤ 0.01 and | log_2_FC| ≥ 2) (Figure 5E). Compared with CK group under the above filter conditions, the LA-2d, LA-7d, HA-2d and HA-7d groups had 236, 33, 679, and 78 differential genes, respectively. There were 211 common genes in LA-2d and HA-2d, 23 common genes in LA-7d and HA-7d, 20 common genes in LA-2d and LA-7d, and 56 common genes in HA-2d and HA-7d. In order to analyze the accuracy of the results, the first 20 independent DEGs were selected from each group, and all the ones less than 20 were selected for KEGG enrichment analysis. According to KEGG enrichment analysis, these genes are mainly enriched in four types of biological metabolic pathways: Cellular Processes, Environmental Information Processing, Metabolism and Organismal Systems (Tables 4, 5).

**TABLE 4:** Effects of AESE dose on DEGs enriched pathways and expression levels.

	Gene ID (ENSRNOG-)	Gene Symbol	Log2FC		Pathway
			LA-2d	HA-2d	
**Cellular Processes**					
	00000003897	*Col1a1*	2.041	2.096	ko04510
	00000020918	*Ccnd1*	3.553	3.511	ko04510
	00000043451	*Spp1*	3.669	5.572	ko04510
	00000053691	*Lama5*	4.817	5.954	ko04510
**Environmental Information Processing**					
	00000051171	*G6pc*	–2.272	–3.933	ko04068, ko04152
	00000020918	*Ccnd1*	3.553	3.511	ko04068, ko04152
	00000021814	*Tnfrsf25*	2.572	3.138	ko04060
	00000000521	*Cdkn1a*	2.646	3.340	ko04068
	00000003546	*Tnfrsf12a*	2.193	2.872	ko04060
	00000007002	*Lif*	2.572	4.111	ko04060
	00000013018	*Eda2r*	2.202	3.829	ko04060
	00000013090	*Gadd45g*		–3.231	ko04068
	00000013552	*Scd*	–5.715	–5.805	ko04152
**Metabolism**					
	00000020420	*Pklr*	–2.539	–3.275	ko01100
	00000020775	*Cyp2b2*	–2.553	–3.338	ko01100, ko00830, ko00590, ko00140
	00000001379	*Cyp3a62*	2.022	2.739	ko01100, ko00830, ko00140, ko00591
	00000001388	*Sds*	–2.858	–5.113	ko00260, ko01100
	00000001466	*LOC100361492*	4.823	5.824	ko01100, ko00830, ko00590, ko00140, ko00591
	00000002597	*Hsd17b6*	–2.134	–2.066	ko01100, ko00830, ko00140
	00000003244	*Ltc4s*	–2.421	–2.386	ko01100, ko00590
	00000005358	*Pmm1*	2.194	2.932	ko01100
	00000009080	*Atp6v1d*	2.604	3.158	ko01100
	00000009734	*Akr1b8*	2.027	3.264	ko01100, ko00561
	00000009754	*Nampt*	–2.115	–2.145	ko01100
	00000009875	*Akr1b7*	2.249	3.880	ko01100, ko00561
	00000010633	*Acsl1*	–2.374	–3.016	ko01100, ko01212, ko00071
	00000011200	*Bhmt*	–2.378	–2.263	ko00260, ko01100
	00000011250	*Inmt*	–4.178	–2.046	ko00380
	00000011381	*Acsbg1*	–2.141	–3.408	ko01100, ko01212, ko00071
	00000011420	*Mtmr7*	–2.012	–2.820	ko01100
	00000013241	*Cyp2c24*	3.631	4.291	ko01100, ko00830, ko00590, ko00140, ko00591
	00000013552	*Scd*	–5.715	–5.805	ko01212, ko01040
	00000015076	*Cyp26b1*	–2.858	–2.474	ko01100, ko00830
	00000016791	*Chka*	–2.533	–2.863	ko01100
	00000016826	*Pla2g2d*	2.266	3.716	ko01100, ko00590, ko00591
	00000016924	*Acly*	–2.206	–3.237	ko01100
	00000017311	*Me3*	–2.881	–3.343	ko01100
	00000017619	*Aldh1a1*	2.654	2.398	ko01100, ko00830
	00000017878	*Aldh1a7*	3.526	4.368	ko01100, ko00830
	00000022268	*Pnpla3*	–2.701	–2.661	ko01100, ko00561
	00000025691	*Pla2g7*	2.749	3.884	ko01100
	00000026764	*Pla2g4c*		–3.987	ko01100, ko00590, ko00591
	00000045743	*Etnppl*	–2.880	–2.627	ko01100
	00000052810	*Cyp2c11*	–3.469	–4.585	ko01100, ko00830, ko00590, ko00140, ko00591
	00000053448	*AC123346.1*	–2.741	–2.936	ko00410, ko00260, ko01100
	00000053691	*Lama5*	4.817	5.954	ko01100
	00000055221	*Acot1*		3.033	ko01100, ko01040
	00000055672	*Gpx2*	2.490	3.264	ko00590
	00000057470	*Pla2g12a*	2.566	2.566	ko01100, ko00590, ko00591
	00000051171	*G6pc*	–2.272	–3.933	ko01100, ko00500
**Organismal Systems**					
	00000005250	*Abcg5*		–3.045	ko04979, ko04975
	00000005420	*Abcg8*	–2.088	–2.148	ko04979, ko04976, ko04975
	00000009686	*Aqp7*	3.037	3.037	ko03320, ko04923
	00000010633	*Acsl1*	–2.374	–3.016	ko03320
	00000010805	*Fabp4*	3.685	5.909	ko03320, ko04923
	00000011381	*Acsbg1*	–2.141	–3.408	ko03320
	00000013552	*Scd*	–5.715	–5.805	ko03320
	00000016826	*Pla2g2d*	2.266	3.716	ko04975
	00000024947	*Fabp2*	2.752	3.916	ko03320, ko04975
	00000051171	*G6pc*	–2.272	–3.933	ko04973
	00000057470	*Pla2g12a*	2.566	2.566	ko04975

			**LA-7d**	**HA-7d**	

**Cellular Processes**					
	00000016148	*Gtse1*	2.691	3.323	ko04115
**Environmental Information Processing**					
	00000001843	*Bcl6*	2.524	2.167	ko04068
	00000013552	*Scd*		–2.671	ko04152
**Organismal Systems**					
	00000010805	*Fabp4*	2.321	3.915	ko03320
	00000013552	*Scd*		–2.671	ko03320

**TABLE 5:** Effects of AESE duration on DEGs enrichment pathway and expression.

	Gene ID (ENSRNOG-)	Gene symbol	Log2FC		Pathway
			**LA-2d**	**LA-7d**	
Cellular processes					
	00000016148	*Gtse1*	3.755	2.691	ko04115
	00000020918	*Ccnd1*	3.553		ko04115
Environmental information processing					
	00000013552	*Scd*	–5.715		ko04152
	00000020918	*Ccnd1*	3.553		ko04068, ko04152
	00000051171	*G6pc*	–2.272		ko04068, ko04152
Organismal systems					
	00000007152	*Bhlhe40*	–2.860	–2.116	ko04710
	00000007545	*Angptl4*	–2.032		ko03320
	00000010633	*Acsl1*	–2.374		ko03320
	00000010805	*Fabp4*	3.685	2.321	ko03320, ko04923
	00000013552	*Scd*	–5.715		ko03320, ko04212
	00000038047	*Mt1*	–2.472	3.249	ko04212
	00000043098	*Mt2A*	–2.189	2.657	ko04212

			**HA-2d**	**HA-7d**	

Environmental information processing					
	00000000521	*Cdkn1a*	3.340	2.015	ko04068
	00000013552	*Scd*	–5.805	–2.671	ko04068
	00000051171	*G6pc*	–3.933		ko04068
Metabolism					
	00000017878	*Aldh1a7*	4.368	2.877	ko00830
	00000019058	*Gstm3l*	4.218	2.875	ko00983
	00000026764	*Pla2g4c*	–3.987		ko00590, ko00591
	00000055672	*Gpx2*	3.264	2.715	ko00590
	00000001466	*LOC100361492*	5.824	3.129	ko00140, ko00590, ko00591, ko00830
	00000005341	*Upp2*		2.226	ko00983
	00000013241	*Cyp2c24*	4.291	2.711	ko00140, ko00590, ko00591, ko00830
Organismal systems					
	00000005794	*Slc10a1*	–4.743		ko04976
	00000010805	*Fabp4*	5.909	3.915	ko03320
	00000013552	*Scd*	–5.805	–2.671	ko03320
	00000024947	*Fabp2*	3.916		ko03320

By analyzing the DEGs in Tables 4, 5, it was found that DEGs of LA-2d and HA-2d were mainly enriched in pathways related to carbohydrate, lipid and amino acid metabolism, while DEGs of LA-7d and HA-7d were mainly enriched in four pathways, which were related to cell cycle regulation, inflammatory response and oxidation reaction. *Lama5*, *Gtse1* and *Fabp4* gene in DEGs were all highly expressed in the model groups. *Bcl6* gene was highly expressed on the 7th day after ASAE, and the expression level was higher than that on the 2nd day after ASAE. *Lama5* gene enrichment in focal adhesion (ko04510) and metabolic pathways (ko01100). Focal adhesion pathway plays an important role in cell motility, cell proliferation, and cell differentiation (Ye et al., 2020). The dysregulation of focal adhesion is considered to be an essential step in tumor invasion, it can promote tumor invasiveness and metastasis (Shen et al., 2018). Metabolic pathway plays an important role in the metabolism of sugar, fat and protein in the liver. Metabolic dysfunction not only affects the normal growth of the body, but also causes various diseases. *Gtse1* gene enrichment in p53 signaling pathway (ko04115). The protein encoded by the *p53* gene is a transcription factor that controls the cell cycle. The occurrence of oxidative stress and DNA damage in the body can cause *p53* mutation, resulting in cell cycle dysregulation (Lacroix et al., 2020; Bailey et al., 2021). *Fabp4* gene enrichment in regulation of lipolysis in adipocytes (ko04923) and PPAR signaling pathways (ko03320). Regulation of lipolysis in adipocytes pathway plays an important role in the dynamic equilibrium of lipid droplet formation and decomposition (Yang and Mottillo, 2020). Dysregulation of this pathway leads to lipid accumulation in the liver causes hepatocyte damage and portal inflammation (An et al., 2021). PPAR signaling pathway plays an essential role in the maintenance of metabolic homeostasis, it can adjust the balance between anabolism and oxidation of adipose tissue to prevent peroxidation (Corrales et al., 2018). *Bcl6* gene enrichment in FoxO signaling pathway (ko04068). The pathway can inhibit cell metabolism, growth, differentiation, oxidative stress, and aging, it is suggested to play a pivotal functional role as a tumor suppressor in cancers (Farhan et al., 2017; Lee and Dong, 2017).

### Quantitative Validation

**FIGURE 6:**
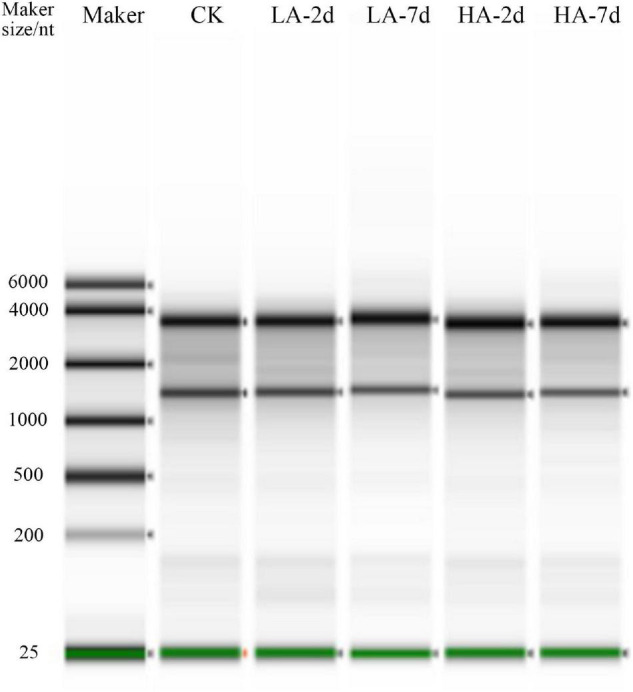
Electrophoresis diagram of the Total RNA Automatic Nucleic Acid Electrophoresis Analyzer.

Quantitative validation of the predicted differential genes based on transcriptome sequencing data was carried out for *Lama5*, *Ccnd1*, *Cdkn1a* and *Gpx2*, which were randomly selected. RNA integrity is shown in the plots from electrophoresis experiments using a fully automated nucleic acid electrophoresis analyzer as in Figure 6. The validation results are shown in Table 6. The transcriptome sequenced genes with Q-PCR showed the same trend in different model groups.

**TABLE 6:** Summary of Q-PCR results and RAN-Seq results for randomly selected genes.

	Fold change								
	CK	LA-2d		LA-7d		HA-2d		HA-7d	
Relative gene expression level		Q-PCR	RNA-Seq	Q-PCR	RNA-Seq	Q-PCR	RNA-Seq	Q-PCR	RNA-Seq
*Lama5*	1	22.91	28.19	13.78	9.93	38.45	61.99	19.19	18.08
*Ccnd1*	1	7.08	11.74	1.25	0	4.88	11.4	2.17	3.07
*Cdkn1a*	1	396.9	6.26	154.39	3.3	446.12	10.13	189.54	4.04
*Gpx2*	1	0.51	5.62	0.39	2.55	0.35	9.61	0.18	6.57

## Discussion

In this study, a rat model of acute injury was established by a single intraperitoneal injection of 1 and 2 mg/kg AFB_1_, and then the rats were fed normally for 2 and 7 days, respectively. The aim was to study the persistent effects of ASAE on rat liver. We found that other indexes except antioxidant enzyme and liver coefficient showed a trend of recovery with the continuous influence time of the AFB_1_ on liver. Among them, ALT, AST and TBIL returned to normal, ALP, hepatocyte apoptosis rate and MDA content still had significant differences compared with CK group (*p* < 0.05). Antioxidant enzyme activity continued to decrease with the prolonged exposure time of AFB_1_. The liver coefficient is rising. Meanwhile, the model was used to explore the persistent effects of acute AFB_1_ exposure on liver genes, and we found that the number of DEGs decreased significantly over time.

The data in this article shows that although there is no significant difference in the weight of rats after ASAE compared with that before ASAE, it still presents a downward trend. It illustrates that AFB_1_ leads to the reduction of food intake in rats, and the decline of the body’s metabolic capacity, so that the nutrients cannot be fully absorbed (Jindal et al., 1994). This is consistent with the results of previous research (Rastogi et al., 2001; Knipstein et al., 2015). Compared with the CK group, SOD and GSH-Px activities decreased, MDA content increased, and cell apoptosis rate increased on the 2nd day after ASAE, indicating that AFB_1_ can cause oxidative stress reaction and liver cell apoptosis, resulting in severe liver damage. This result is similar to other published papers on acute liver injury caused by AFB_1_ (Wang et al., 1991; Kumagai et al., 1998; Benkerroum, 2020; Ruggeberg et al., 2020). On the 4th day after ASAE, ALT and AST values decreased, but they were still significantly higher than the CK group (*p* < 0.05), which was consistent with previous studies (Monmeesil et al., 2019). At the same time, we found that ALT, AST, and TBIL returned to normal on day 7th after ASAE, but other phenotypic indexes still showed significant differences compared with the CK group. However, compared with the 2nd day after ASAE, the phenotypic indexes except antioxidant enzymes and liver coefficient showed significant differences, but showed a recovery trend. These results showed that AFB_1_ still caused rats liver damage to a certain extent after feeding for 7 days after ASAE. The recovery of liver function indexes ALT, AST and TBIL may be related to the strong self-repair ability of liver (Ko et al., 2020), and the change trend of apoptosis rate and MDA content also indicates that the compensatory function of liver plays a role (Hall et al., 2021). The ALP value is related to whether the intrahepatic and extrahepatic bile ducts are blocked. The ALP value on the 7th day after ASAE was still significantly higher than that of the CK group, this is related to the fact that cholestasis caused by bile duct blockage does not self-adjust and recover in a short time (Boyer, 2013; Taylor et al., 2020). The changes of pathological section and liver coefficient further indicated that liver morphological damage was difficult to recover spontaneously in a short time. The antioxidant enzymes GSH-Px and SOD activities continued to decrease with the prolongation of AFB_1_ influence time. On the 2nd day after ASAE, the change trend of antioxidant enzyme activity was similar to that reported in the previous paper (Deng et al., 2020). The changes of antioxidant enzymes on the 7th day after ASAE may be related to the fact that the liver damage of rats is not alleviated and the intake of antioxidant nutrients is insufficient due to decreased appetite (Michiels et al., 1994; Peskin, 1997; Liochev and Fridovich, 2010). However, the reasons for the changes in lipid peroxide content and antioxidant enzyme activity in this model need to be further explored through related experiments.

**FIGURE 7:**
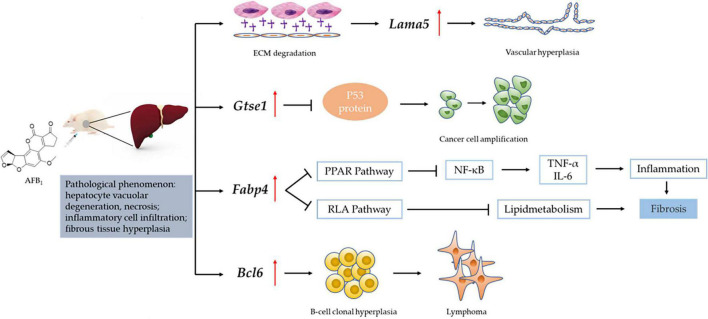
Mechanism of liver injury induced by *Lama5*, *Gtse1*, *Fabp4*, and *Bcl6* genes. RAF: Regulation of lipolysis in adipocytes; ECM: Extracellular matrix; “ 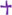 “: vascular endothelial growth factor (VEGF); “ 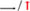 “: promote/up-regulate; “ 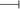 “: inhibition.

We obtained the gene data of each experimental group by RNA-Seq technique and found that the number of DEGs was affected by the dose and duration of AFB_1_ exposure, among which *Lama5*, *Gtse1*, *Fabp4* and *Bcl6* genes were continuously affected and associated with liver injury. Their mechanism of action is shown in Figure 7. *Lama5* gene plays a role in promoting angiogenesis during tumor development and is a key promoter of liver metastatic growth (Yousif et al., 2013; Gordon-Weeks et al., 2019). Tumor-infiltrating myeloid cells can drive tumor cells to express *Lama5* through *NF-*κ*B* gene transduction, thereby promoting angiogenesis (Bonnans et al., 2014). The Lama5 protein chain is an important element of the extracellular matrix (ECM). Thus, the *Lama5* gene links two markers of cancer progression (Inflammation and Angiogenesis) to extracellular matrix (ECM) protein deposition. Related studies had shown that down-regulation of *Lama5* can reduce the formation of vascular branches, and even tend to normalize (Carmeliet and Jain, 2011; Di Russo et al., 2017). *Fabp4* has also been shown to be involved in inflammatory responses. Cytokine levels, including TNF-α and IL-1β, were significantly increased in macrophages with high *Fabp4* expression (Makowski et al., 2001). And *Fabp4* plays an important role in transporting fatty acids between fat cells and cancer cells (Besnard et al., 2002). These effects ultimately may lead to fibrosis of tissues (Nieman et al., 2011). The proto-oncogene *Bcl6* is an important transcriptional regulator of the immune system, and its upregulation is a marker of B cell accumulation through the germinal center (GC) transport process. In tumors, its dysregulated expression may promote lymphoma by increasing B cell resistance to apoptosis and inhibiting B cell differentiation in the GC (Murakami et al., 1999; Niu, 2002). Low-grade B-cell lymphomas can occur in the liver and manifest as a characteristic massive infiltration of lymphocytes within the confluent zone. *Lama5*, *Fabp4* and *Bcl6* gene related to inflammation and immune response were all up-regulated, and the expression of *Bcl6* gene was higher at 7 days than at 2 days after ASAE. Their changing trend explained that the pathological phenomena of liver was not relieved the 7th day after ASAE to some extent. And there were still different degrees of liver cell necrosis, inflammatory cell infiltration and fibrous tissue hyperplasia. *Gtse1* has been found to be overexpressed in a variety of cancers (Tian et al., 2011). Previous studies have shown that it inhibits apoptosis induced by the tumor suppressor protein p53 (Liu et al., 2010). Cell level experiments showed that *Gtse1* was down-regulated, the protein content of p53 in cells increased, and the dephosphorylation of protein kinase B and cyclin B1 decreased, thus inhibiting the proliferation of HCC cells and promoting apoptosis (Guo et al., 2016). Our data showed that, the expression level of *Gtse1* on the 7th day after ASAE was lower than that on the 2nd day after ASAE, which may explain why the apoptosis rate of liver cells decreased on the 7th day after ASAE. However, the specific regulatory mechanism needs further study.

## Conclusion

In summary, ASAE can cause persistent liver damage. The experiment period of this study was 7 days, and the damage of liver during the extended experiment period could be further studied. The persistently affected *Lama5*, *Gtse1*, *Fabp4* and *Bcl6* gene can be potential target genes for the treatment of AFB_1_ induced liver diseases, but their effectiveness needs to be further explored in experiments.

## Data Availability Statement

The data presented in the study are deposited in the NCBI repository, accession number BioProject ID PRJNA823496.

## Ethics Statement

The animal study was reviewed and approved by Shanghai University of Traditional Chinese Medicine.

## Author Contributions

JY: conceptualization, methodology, formal analysis, investigation, resources, data curation, writing—original draft preparation, and visualization. LC: methodology, formal analysis, and resources. LZ: investigation and resources. ZZ: validation, writing—review and editing and funding acquisition. YZ: supervision, project administration, and funding acquisition. YW: conceptualization, validation, and project administration. JO: conceptualization, validation, writing—review and editing, supervision, and project administration. All authors contributed to the article and approved the submitted version.

## Conflict of Interest

The authors declare that the research was conducted in the absence of any commercial or financial relationships that could be construed as a potential conflict of interest.

## Publisher’s Note

All claims expressed in this article are solely those of the authors and do not necessarily represent those of their affiliated organizations, or those of the publisher, the editors and the reviewers. Any product that may be evaluated in this article, or claim that may be made by its manufacturer, is not guaranteed or endorsed by the publisher.
